# Experiential avoidance and smartphone addiction in adolescents: a study using multi-model machine learning and mediation analysis

**DOI:** 10.3389/fpsyg.2026.1860144

**Published:** 2026-10-06

**Authors:** Xinyao Zhang, Weixin Zhang

**Affiliations:** 1 Department of Social Medicine and Health Management, School of Public Health, Jilin University, Changchun, Jilin, China; 2 Infectious Disease Prevention and Control Department, Xi'an New District Center for Disease Control and Prevention, Xi'an, Shaanxi, China

**Keywords:** adolescents, cross-sectional study, experiential avoidance, machine learning, smartphone addiction, social support

## Abstract

**Background:**

Smartphone addiction is closely associated with adolescents’ mental health and educational outcomes. Current research lacks comprehensive machine learning studies for predicting smartphone addiction factors, and studies examining relationships between various factors including mediation effects are also insufficient.

**Methods:**

A cross-sectional survey was conducted among 3,405 adolescents in China (1,853 boys, 54.4%; 1,552 girls, 45.6%; mean age = 14.50 years, SD = 1.18). Eight machine learning algorithms (Linear Regression, Ridge Regression, Random Forest, XGBoost, LightGBM, CatBoost, Support Vector Regression, Bayesian Ridge Regression) were employed with SHAP analysis for interpretability. Predictive variables included demographic characteristics, social support, experiential avoidance, self-efficacy, and emotional symptoms. Baron and Kenny’s method was used for mediation effect analysis.

**Results:**

Linear models demonstrated optimal performance (R^2^ = 0.478, RMSE = 8.66). SHAP analysis identified experiential avoidance as the strongest predictor (SHAP value = 5.472), followed by depressive symptoms (1.723), BMI category (1.438), age (1.394), and anxiety symptoms (1.337). Mediation analysis revealed that social support’s negative association with smartphone addiction (total effect = −0.231) was almost indirect (−0.252), with experiential avoidance mediating the majority of the total effect. The final model explained 49.4% of variance in smartphone addiction.

**Conclusion:**

Experiential avoidance is a strong correlate of adolescent smartphone addiction and plays a mediating role in the association between social support and smartphone addiction. This finding is consistent with the “reality deficit-virtual compensation” theoretical framework. Additionally, depressive symptoms, BMI category, age, and anxiety symptoms were also independently associated with smartphone addiction.

## 1 Introduction

Smartphone ownership has increased dramatically across the globe, with over 6.4 billion users projected by 2029, predominantly among young individuals. A recent meta-analysis indicates that approximately 1 in 4 children and young people (CYP) are demonstrating problematic smartphone use, a pattern of behavior that mirrors that of a behavioral addiction (Sohn et al., 2019), with a median prevalence of 23.3%. Smartphone addiction, or problematic smartphone use (PSU), is characterized by tolerance, withdrawal symptoms, preoccupation, neglect of other activities, subjective loss of control, and continued use despite evidence of harm. A meta-analysis across 24 countries, 83 samples, and 33,831 participants demonstrates that problematic smartphone use is increasing across the world, with China, Saudi Arabia, and Malaysia having the highest scores (Olson et al., 2022). In China specifically, research indicates that more than 25% of Chinese adolescents experience smartphone addiction (Zhang et al., 2025), with some regional studies reporting even higher rates of 32.5% in certain areas. The consequences of smartphone addiction on adolescents are multifaceted and severe. PSU was associated with an increased odds of depression, increased anxiety, higher perceived stress, and poorer sleep quality (Sohn et al., 2019). Academic performance is also impacted, with studies showing consistent associations between PSU and poor educational attainment. The gravity of this issue is underscored by emerging evidence linking smartphone addiction to physical health problems, including hypertension among adolescents. This represents an urgent public health challenge requiring immediate attention and evidence-based interventions.

Traditional statistical methods, while valuable, face limitations when dealing with the complex, non-linear relationships inherent in addiction behaviors. Machine learning algorithms offer superior predictive accuracy and pattern recognition capabilities that can uncover hidden relationships in multidimensional data. SHAP (SHapley Additive exPlanations), a popular feature-based interpretability method, can be seamlessly integrated into supervised ML models to gain a deeper understanding of their predictions, thereby enhancing their transparency and trustworthiness (Ponce-Bobadilla et al., 2024). A recent study using SHAP-based explainable ML framework for Internet addiction prediction in children and adolescents achieved excellent performance, with key predictors including grade level, delinquent behavior, anxiety, family function, and depression scores (Liu et al., 2025). The SHAP methodology provides both global feature importance through mean absolute SHAP values and local explanations for individual predictions, enabling consistent comparison of feature contributions across different model types. SHAP assigns each feature an importance value for a particular prediction, with its novel components including the identification of a new class of additive feature importance measures, and theoretical results showing there is a unique solution in this class with a set of desirable properties (Lundberg and Lee, 2017). Despite these advantages, current research employing multi-model comparison with SHAP-based interpretability in the context of adolescent smartphone addiction remains limited, indicating a critical gap that needs to be addressed to advance our understanding of key risk factors.

Previous research has consistently demonstrated that adolescents lacking social support in real life are more prone to excessive internet and smartphone use. The virtual world provides the sense of belonging and identity that is missing in their real-world interactions, creating a compensatory mechanism for social deficits (Kardefelt-Winther, 2014). Cullen’s Social Support Theory posits that social support refers to the information individuals obtain from social relationships, leading them to believe that they are cared for, loved, respected, and valued, and that they belong to a reciprocal social network (Cui et al., 2024). Multiple studies have documented the correlation between social isolation and excessive internet use, with studies on Chinese adolescent samples reporting a significant negative correlation between perceived social support and Internet addiction (Cui et al., 2024). Social media and online gaming platforms become tools for compensating real-world social deficits, providing immediate gratification and virtual connections that temporarily alleviate feelings of loneliness and isolation. People with experiential avoidance do not have the opportunity to establish and maintain interpersonal relationships or experience meaningful relationships, so it is difficult to experience close interpersonal relationships (Kim and Bae, 2022). However, the complete pathway from social support deficiency → increased experiential avoidance → smartphone addiction has rarely been tested within a single model in a large adolescent sample.

Experiential avoidance is defined as the unwillingness to remain in contact with aversive internal experiences such as painful feelings, thoughts, and emotions. Experiential avoidance (EA) refers to an intentional behavior to avoid or escape from aversive internal experiences such as affect, thoughts, memories, and bodily sensations among private events (Na et al., 2022). Within the Acceptance and Commitment Therapy (ACT) theoretical framework, the ACT approach posits that psychological human suffering is due to a lack of behavioral flexibility and effectiveness, which emerges from experiential avoidance, cognitive entanglement, difficulty with perspective taking, loss of contact with the present, and failure to take needed behavioral steps in accord with core values (Dindo et al., 2017). Various studies have reported the adverse consequences of destructive and rigid EA, which are reflected in transdiagnostic mental illnesses such as depressive disorders, anxiety disorders, obsessive-compulsive disorder, substance use disorder, eating disorders, and post-traumatic stress disorder (Na et al., 2022). Experiential avoidance has been linked to a range of addictive behaviors, including technological addictions in adolescents (Garcia-Oliva and Piqueras, 2016), and recent studies of Chinese adolescents have identified it, together with depressive or anxiety symptoms, as a mediator linking adverse social experiences to smartphone addiction (Lin et al., 2023; Xiong et al., 2023). Smartphone use serves as a readily available avoidance strategy, providing immediate distraction from uncomfortable internal experiences through endless scrolling, gaming, or social media engagement. Better distress tolerance and lower experiential avoidance were negatively and significantly associated with alcohol and drug cravings, but only experiential avoidance remained significantly and negatively associated with cravings when both predictors were examined simultaneously (Shorey et al., 2017). Experiential avoidance may thus represent the critical bridge connecting psychological distress with addictive behaviors, as individuals increasingly rely on smartphones to escape rather than confront their internal experiences.

Social support represents a multidimensional construct encompassing emotional, instrumental, and informational support from family, peers, and school systems, and is a key resource for adolescent resilience (Çiçek, 2021). Strong religious beliefs integrated into society serve as a crucial protective factor that can prevent adolescents from engaging in drug abuse, in addition, the school connectedness and adult support also play a major contribution in the drug use (Nawi et al., 2021). Social support has been extensively researched in the context of recovery from substance use disorders, with past research noting a positive correlation between social support and recovery, higher quality of life, subjective well-being, days of abstinence, less stress, and treatment retention (Islam et al., 2023). The protective effects of social support operate through multiple mechanisms, including buffering against stress, enhancing coping abilities, resilience and self-efficacy (Green et al., 2024), and providing alternative sources of reward and reinforcement. Social support has a positive effect in maintaining the physical, emotional, and mental health of drug abusers, reducing the tendency to use illicit drugs (Liu et al., 2022). Research has shown significant negative correlations between perceived social support and addiction severity (Cui et al., 2024). Baseline experiential avoidance mediated the association between lifetime PTSD symptoms and 8-month social support (Kelly et al., 2019), suggesting that social support deficits may increase vulnerability to experiential avoidance. However, while the direct protective effects of social support are well-established, the specific mechanisms through which social support is linked to lower experiential avoidance and, in turn, to less smartphone addiction remain inadequately understood.

Depression and anxiety are highly prevalent among adolescents, with rates continuing to rise globally. A previous meta-analysis of mobile phone addiction in children and adolescents by Sohn et al. found that adolescents in the 17–19 age group exhibited a higher prevalence of mobile phone addiction (Sohn et al., 2019). Negative emotional states are associated with greater smartphone addiction in adolescents, and psychological resources such as resilience can attenuate this link (Yıldırım and Çiçek, 2022). The bidirectional relationship between negative emotions and smartphone addiction is well-documented, with longitudinal studies suggesting reciprocal relationships. Adolescents with depressive symptoms report higher levels of mobile phone addiction (Luo et al., 2025). Experiential avoidance and interpersonal problems completely mediate the relationship between social anxiety and SNS addiction proneness sequentially (Kim and Bae, 2022). Emotional symptoms may function as serial mediators in the pathway from social support deficiency to smartphone addiction, operating through experiential avoidance. Specifically, lack of social support may be accompanied by depressive and anxious symptoms, which in turn may heighten experiential avoidance tendencies as individuals struggle to cope with overwhelming negative emotions, with smartphone use ultimately serving as an escape mechanism.

Taken together, these strands of evidence can be integrated into a “reality deficit–virtual compensation” framework. Drawing on compensatory internet use theory (Kardefelt-Winther, 2014), adolescents whose need for connection and support is unmet in everyday life are expected to turn to the smartphone to compensate; drawing on the ACT model of psychological inflexibility (Dindo et al., 2017), this compensation is expected to operate largely through experiential avoidance, because the smartphone offers an immediate means of escaping the negative emotions that accompany a lack of support. On this basis we hypothesized that (H1) experiential avoidance is the strongest correlate of smartphone addiction among the psychosocial and demographic variables examined; (H2) perceived social support is negatively associated with smartphone addiction; (H3) experiential avoidance mediates the association between social support and smartphone addiction; and (H4) depressive and anxiety symptoms mediate this association both directly and serially through experiential avoidance.

The present study aims to: (1) employ multiple machine learning algorithms for comparative analysis to identify key predictors of adolescent smartphone addiction, utilizing SHAP methodology to provide interpretable insights into feature importance and interactions; (2) systematically examine whether the association between social support and smartphone addiction is mediated by experiential avoidance (including the serial mediating roles of depression and anxiety). This integrated approach combining advanced machine learning techniques with theory-driven mediation analysis will provide comprehensive empirical evidence for understanding the psychological processes associated with adolescent smartphone addiction, thereby informing the development of targeted, evidence-based intervention strategies.

Although experiential avoidance has been examined as a mediator in smaller samples with other antecedents, such as harsh parenting (Lin et al., 2023) or negative school gossip (Xiong et al., 2023), three questions remain open: how much experiential avoidance matters relative to emotional symptoms, social support, self-efficacy and demographic characteristics when all are considered simultaneously; whether non-linear models add predictive value over linear ones for these psychosocial variables; and whether social support is linked to smartphone addiction through experiential avoidance alone or also through parallel and serial emotional pathways. The present study addresses these questions in a large school-based sample by combining a multi-model comparison with SHAP-based importance ranking and a theory-driven serial mediation model.

## 2 Methods

### 2.1 Study population and recruitment strategy

A cross-sectional investigation was implemented in Xi’an, Shaanxi Province, China, spanning the period from March through June 2025. The study employed a cluster-based randomized sampling approach to identify 12 secondary schools from the municipal educational database. All students officially enrolled in these institutions were approached for participation, encompassing a total population of 3,618 adolescents. The complete enrollment records were obtained from the administrative offices of the selected schools. An online survey platform facilitated data collection through school-integrated digital systems. Among the eligible population, 3,433 individuals successfully submitted completed surveys, resulting in a participation rate of 94.88%. Responses were anonymous, participants were told that there were no right or wrong answers, and the scales were presented in separate sections of the questionnaire.

Eligibility criteria for inclusion comprised: (1) age range of 12–18 years; (2) active enrollment status in grades 7 through 12; (3) sufficient comprehension skills to independently respond to questionnaires in Chinese; and (4) documented consent from both the participants and their legal guardians or parents. Exclusion criteria encompassed: (1) presence of serious psychiatric conditions necessitating professional mental health intervention; (2) cognitive limitations that could compromise questionnaire comprehension; or (3) substantially incomplete survey responses with critical data gaps.

The research protocol received ethical clearance from the Institutional Review Board of Jilin University, Changchun, China (2023-11-08). Prior to data collection, written authorization was secured from all study participants along with their parental or guardian consent. The investigation adhered to all ethical principles outlined in the Declaration of Helsinki.

### 2.2 Study instruments

Given the complex and multidimensional characteristics of smartphone addiction among adolescents, our investigation gathered extensive sociodemographic data covering both personal attributes and household factors. Personal characteristics assessed included biological sex (male or female), chronological age (measured in years), single-child household status (affirmative or negative), Body Mass Index classification (categorized as underweight, normal weight, overweight, or obese; World Health Organization, 2010), and subjective assessment of physical health condition (rated as poor, moderate, or good). Household-related factors examined included parental educational attainment (classified as secondary education or less, undergraduate degree, or postgraduate degree and higher) and household composition (categorized as 2 or fewer members, 3 members, 4 members, or 5 and above members).

Smartphone addiction was evaluated using the Mobile Phone Addiction Index (MPAI), originally developed by Leung (2008) and subsequently validated by Huang et al. (2014). This instrument comprises 17 items distributed across four dimensions: inability to control craving, feeling anxious and lost, withdrawal or escape, and productivity loss. Sample items include “You find yourself engaged on the mobile phone for longer periods of time than intended” and “You feel lost without your mobile phone”. Each item is rated on a five-point scale ranging from 1 (never) to 5 (always), yielding a total score between 17 and 85. Higher scores indicate greater levels of smartphone addiction. The Cronbach’s α for this scale was 0.89.

Social support was assessed using the Chinese adaptation of the Perceived Social Support Scale (PSSS), originally developed by Zimet et al. (1988) and adapted for Chinese populations (Li and Qin, 2020). This 12-item instrument measures two primary dimensions: family support and support from outside the family, utilizing a 7-point Likert response format (1 = strongly disagree, 7 = strongly agree). Sample items include “My family really tries to help me” and “I can count on my friends when things go wrong”. Total scores range from 12 to 84, with higher scores indicating greater perceived social support. The Cronbach’s α for this scale was 0.86.

Experiential avoidance was measured using the Acceptance and Action Questionnaire-II (AAQ-II; Fledderus et al., 2012), validated for Chinese populations by Cao et al. (2013). This 7-item scale employs a 7-point Likert response format (1 = never, 7 = always), generating total scores from 7 to 49. Sample items include “I’m afraid of my feelings” and “My painful memories prevent me from having a fulfilling life”. Higher scores reflect greater levels of experiential avoidance among participants. The Cronbach’s α for this scale was 0.87.

Self-efficacy was measured through the Chinese version of the General Self-Efficacy Scale (GSES), originally constructed by Schwarzer and Jerusalem, 1995. This 10-item scale utilizes a 4-point Likert format (1 = not at all true, 4 = exactly true), producing scores ranging from 10 to 40. A sample item is “I can always manage to solve difficult problems if I try hard enough”. Higher scores represent stronger self-efficacy beliefs. The Cronbach’s α for this scale was 0.937.

Negative emotional states were assessed using the Depression Anxiety Stress Scales-21 (DASS-21), developed by Lovibond and Lovibond (1995) and validated in Chinese by Cao et al. (2023). This widely-utilized 21-item measure encompasses three subscales of 7 items each, measuring depression, anxiety, and stress symptoms. Items are rated on a 4-point scale reflecting the frequency of symptoms over the past week. Sample items include “I felt that I had nothing to look forward to” (depression) and “I felt I was close to panic” (anxiety). Higher scores indicate more severe psychological distress. Subscale scores were multiplied by two to make them comparable with the full DASS-42, following the scale authors’ recommendation. For classification purposes, cutoff scores were established as ≥10 for depression, ≥8 for anxiety, and ≥15 for stress symptoms. The Cronbach’s α for this scale was 0.909. Binary variables were coded 0/1 (gender: 0 = female, 1 = male; only-child status: 0 = no, 1 = yes; depressive, anxiety and stress symptoms: 0 = below the cut-off, 1 = at or above the cut-off).

### 2.3 Statistical analysis

Prior to conducting the main analyses, data quality was assessed through outlier detection using box-plot methods to identify and handle extreme values that could potentially bias the results. Descriptive statistical analyses were then performed to characterize the distribution of all study variables. For continuous variables, measures of central tendency (mean and median) and dispersion (standard deviation, variance, and coefficient of variation) were calculated to provide a comprehensive overview of the data distribution patterns and variability within the sample.

To examine the associations between smartphone addiction and various predictors, univariate analyses were conducted using different statistical approaches based on the nature and distribution of the variables. For continuous variables, Pearson correlation analysis was employed when data met normality assumptions, while Spearman rank correlation was utilized for non-normally distributed variables. For categorical variables, independent samples t-tests were used to compare smartphone addiction scores between two groups, and one-way analysis of variance (ANOVA) was applied for comparisons across multiple groups when parametric assumptions were satisfied. In cases where data violated parametric assumptions, the non-parametric Kruskal-Wallis test was employed as an alternative. Additionally, correlation matrices using either Pearson or Spearman coefficients were constructed to examine the interrelationships among all continuous variables, providing insights into potential multicollinearity and variable clustering patterns that would inform subsequent multivariate analyses.

Because all variables were self-reported in a single survey, common method bias was assessed with Harman’s single-factor test: all 67 items of the five scales were entered into an unrotated principal component analysis, and the proportion of variance explained by the first component was examined, with a value below 40% taken to indicate that common method variance was not a serious concern (Podsakoff et al., 2003).

Feature engineering procedures included encoding categorical variables using one-hot encoding for nominal variables and label encoding for ordinal variables. Continuous variables were standardized using z-score normalization to ensure comparability across different measurement scales. Multicollinearity diagnostics were performed by calculating variance inflation factors (VIF) for all predictors, with a threshold of VIF < 10 applied to identify problematic variables. The dataset was partitioned into training (70%) and testing (30%) sets using stratified random sampling to maintain the distribution of the outcome variable.

Multiple linear regression served as the baseline model, with prerequisite assumptions of linearity, independence, homoscedasticity, and normality verified prior to implementation. The model outputs included regression coefficients, standard errors, t-values, *p*-values, and 95% confidence intervals for each predictor. Ridge regression was employed to address multicollinearity issues, with the regularization parameter α optimized through 5-fold cross-validation to balance bias-variance tradeoff.

Tree-based ensemble methods included Random Forest, XGBoost, LightGBM, and CatBoost. Random Forest regression was configured with 500 trees, maximum depth of 15, and minimum samples for splitting set at 20, with feature importance assessed using both Gini importance and permutation importance metrics. XGBoost was implemented with a learning rate of 0.1, 300 estimators, and maximum depth of 6, incorporating both L1 and L2 regularization terms to prevent overfitting.

LightGBM was selected for its computational efficiency and ability to directly handle categorical features without preprocessing, using histogram-based algorithms for faster training. CatBoost was included specifically for its automatic categorical variable processing through target encoding, eliminating the need for manual categorical encoding while reducing overfitting risk through ordered boosting.

Support Vector Regression (SVR) was applied with standardized input features, testing three kernel functions: RBF, linear, and polynomial. Hyperparameters C (regularization parameter), epsilon (margin of tolerance), and gamma (kernel coefficient) were optimized through grid search with 5-fold cross-validation. Bayesian Ridge Regression was implemented with automatic relevance determination (ARD) to provide probabilistic predictions with uncertainty quantification, allowing for assessment of prediction confidence intervals.

Model performance was assessed using multiple regression metrics. Goodness-of-fit was evaluated through R^2^ and Adjusted R^2^ to account for model complexity. Prediction errors were quantified using Root Mean Square Error (RMSE) for sensitivity to large errors, Mean Absolute Error (MAE) for average prediction accuracy, and Mean Absolute Percentage Error (MAPE) for scale-independent error assessment. Residual analysis included examination of residual plots to identify patterns, Q-Q plots for normality assessment, and Breusch-Pagan tests for heteroscedasticity detection.

Model comparisons were conducted using statistical tests to determine significant differences in predictive performance. The Diebold-Mariano test was applied to compare forecast accuracy between non-nested models. F-tests were used for nested model comparisons to assess whether additional complexity improved model fit. Information criteria including Akaike Information Criterion (AIC) and Bayesian Information Criterion (BIC) were calculated for model selection, balancing goodness-of-fit with model parsimony. Bootstrap resampling with 1,000 iterations was performed to construct 95% confidence intervals for all performance metrics, providing robust estimates of model uncertainty.

SHapley Additive exPlanations (SHAP) analysis was applied as a unified framework for model interpretation across all algorithms. SHAP values were calculated for each feature in every model, providing both global feature importance through mean absolute SHAP values and local explanations for individual predictions. This approach enabled consistent comparison of feature contributions across different model types, from linear models to complex ensemble methods.

Multi-model importance rankings were compiled by aggregating feature importance scores across all eight algorithms, identifying predictors that consistently demonstrated strong associations with smartphone addiction regardless of the modeling approach. Feature selection consistency was evaluated by examining the overlap of top-ranked features across different models, with features appearing in the top 10 importance rankings of at least 6 out of 8 models considered as stable predictors. This consensus approach provided robust identification of core risk and protective factors for smartphone addiction.

This study constructs a multi-path mediation model based on theoretical literature to examine how social support is related to mobile phone addiction. According to the research hypotheses, perceived social support is associated with adolescent mobile phone addiction through experiential avoidance as the core mediating variable. Considering the important role of emotional factors in addictive behaviors, the model also incorporates depression and anxiety as serial mediators, forming three theoretical pathways: the main path where social support is linked to mobile phone addiction through experiential avoidance; parallel path one where social support is linked to mobile phone addiction through the serial mediation of anxiety and experiential avoidance; and parallel path two where social support is linked to mobile phone addiction through the serial mediation of depression and experiential avoidance. Additionally, the model considers the direct effects of anxiety and depression on mobile phone addiction to comprehensively reveal the complex relationships among variables.

The mediation effect testing employs the stepwise regression method, a classical approach widely used in social science research. This method systematically verifies mediation mechanisms through four progressive steps: first, testing whether the total effect of the independent variable on the dependent variable is significant; second, examining the predictive effect of the independent variable on the mediator; third, testing the mediator’s effect on the dependent variable while controlling for the independent variable; and finally, examining the significance and changes in the direct effect when both the independent variable and mediator are included simultaneously. Through hierarchical regression analysis, control variables (age, gender, only-child status, BMI category, parents’ education level, family size and self-rated physical health), independent variables, and mediators are added stepwise, and by comparing changes in R^2^ and regression coefficients at each step, the existence and type of mediation effects can be determined. When the direct effect becomes non-significant after adding the mediator, it indicates full mediation; when it remains significant but the effect size decreases, it indicates partial mediation. Throughout, the terms total, direct and indirect effect are used in their conventional path-analytic sense; because the data are cross-sectional, they describe statistical associations and do not imply causation.

### 2.4 Statistical analysis software and implementation

All statistical analyses in this study were performed using Python 3.8. Data processing and machine learning models were implemented using pandas, NumPy, scikit-learn, XGBoost, LightGBM, and CatBoost libraries, while SHAP was used for model interpretability analysis. Mediation effects were tested through hierarchical regression with bootstrap confidence intervals (5,000 iterations).

## 3 Results

Prior to the main analysis, outlier detection was conducted on all continuous variables using box-plot methods to ensure data quality and identify extreme values that could potentially bias the results. Supplementary Figure 1 presents the box-plot distributions for key study variables. Through this systematic outlier screening process, 28 cases were identified as containing extreme values across multiple variables. These outliers, representing data points beyond 1.5 times the interquartile range from the first and third quartiles, were removed from the dataset. Consequently, the final analytical sample comprised 3,405 adolescents, reduced from the initial 3,433 participants, yielding a retention rate of 99.18%. The box-plots reveal that after outlier removal, all variables demonstrated relatively symmetrical distributions with minimal skewness, suggesting the appropriateness of the cleaned dataset for subsequent parametric analyses.

Table 1 presents descriptive statistics and correlations for continuous variables. The Mobile Phone Addiction Index averaged 52.70 (SD = 12.07), indicating moderate to high addiction levels among participants with a mean age of 14.50 years (SD = 1.18). Experiential avoidance showed the strongest positive correlation with mobile phone addiction (*r* = 0.625, *p* < 0.001), while perceived social support (Mean = 46.12, SD = 10.51) demonstrated a significant negative correlation (*r* = −0.243, *p* < 0.001). Age was weakly positively correlated with addiction (r = 0.154, *p* < 0.001), whereas general self-efficacy (Mean = 26.36, SD = 5.76) showed no significant association (*r* = 0.018, *p* = 0.304).

**Table 1:** Descriptive analysis of continuous variables.

Variable	Mean	Median	Std	Variance	CV	Pearson r	Pearson *p*	Spearman r	Spearman *p*
Age	14.496	14.000	1.178	1.389	8.129	0.154	< 0.001	0.156	< 0.001
General self-efficacy score	26.361	26.000	5.755	33.123	21.833	0.018	0.304	0.009	0.587
Perceived social support score	46.115	46.000	10.506	110.383	22.783	−0.243	< 0.001	−0.247	< 0.001
Experiential avoidance score	30.730	31.000	9.084	82.515	29.560	0.625	< 0.001	0.603	< 0.001
Mobile phone addiction index	52.703	53.000	12.066	145.588	22.894	NA	NA	NA	NA

Pearson r and Spearman r refer to the correlation with the Mobile Phone Addiction Index.

Table 2 presents the comparison of mobile phone addiction scores across categorical variables. Significant differences were found for gender (t = 3.991, *p* < 0.001), with females (M = 53.60, SD = 11.90) showing higher addiction levels than males (M = 51.95, SD = 12.16). BMI category showed significant variation (*F* = 23.575, *p* < 0.001), with obese adolescents displaying the highest addiction scores (M = 56.16, SD = 11.58) compared to underweight participants (M = 50.01, SD = 12.08). Depression and anxiety symptoms were strongly associated with addiction levels: adolescents with depressive symptoms scored significantly higher (M = 57.58, SD = 11.03) than those without (M = 49.78, SD = 11.71, t = −19.218, *p* < 0.001), while those with anxiety symptoms showed higher addiction scores (M = 55.58, SD = 11.58) than those without anxiety symptoms (M = 48.78, SD = 11.61, t = −16.926, *p* < 0.001). Figure 1 displays the correlation matrix examining relationships among all study variables. Most demographic variables demonstrated minimal intercorrelations (|r| < 0.03), suggesting relatively independent contributions to the model. The overall pattern of low correlations among predictors indicates minimal multicollinearity concerns for subsequent regression analyses.

**Table 2:** Descriptive analysis of categorical variables.

Variable	Group	Sample size (n)	Mean (M)	Standard deviation (SD)	t/F	*p*
Gender	Female	1,552	53.603	11.897	3.991	< 0.001
	Male	1853	51.950	12.158		
Parents’ education level	Secondary School or below	862	52.304	12.592	1.784	0.168
	Bachelor’s Degree	2,101	53.005	11.831		
	Master’s degree and above	442	52.048	12.102		
Only child	Non-only child	2,174	52.039	11.952	−4.283	< 0.001
	Only child	1,231	53.877	12.181		
Physical health status	Poor	558	52.667	12.548	0.972	0.378
	Average	1741	52.962	11.973		
	Good	1,106	52.316	11.964		
BMI category	Underweight	262	50.008	12.081	23.575	< 0.001
	Normal	2,156	51.939	12.074		
	Overweight	457	53.847	11.688		
	Obese	530	56.160	11.581		
Family size	2 or fewer people	670	52.151	12.562	1.341	0.259
	3 people	1,241	53.060	12.045		
	4 people	1,036	52.909	11.851		
	5 or more people	458	52.079	11.848		
Depressive symptoms	No depressive symptoms	2,128	49.779	11.713	−19.218	< 0.001
	Depressive symptoms present	1,277	57.576	11.030		
Anxiety symptoms	Anxiety symptoms present	1965	55.581	11.575	16.926	< 0.001
	No anxiety symptoms	1,440	48.776	11.610		

t statistics (independent-samples t-tests) were computed as the mean of the first-listed group minus the mean of the second-listed group; F statistics are from one-way ANOVA.

**Figure 1:**
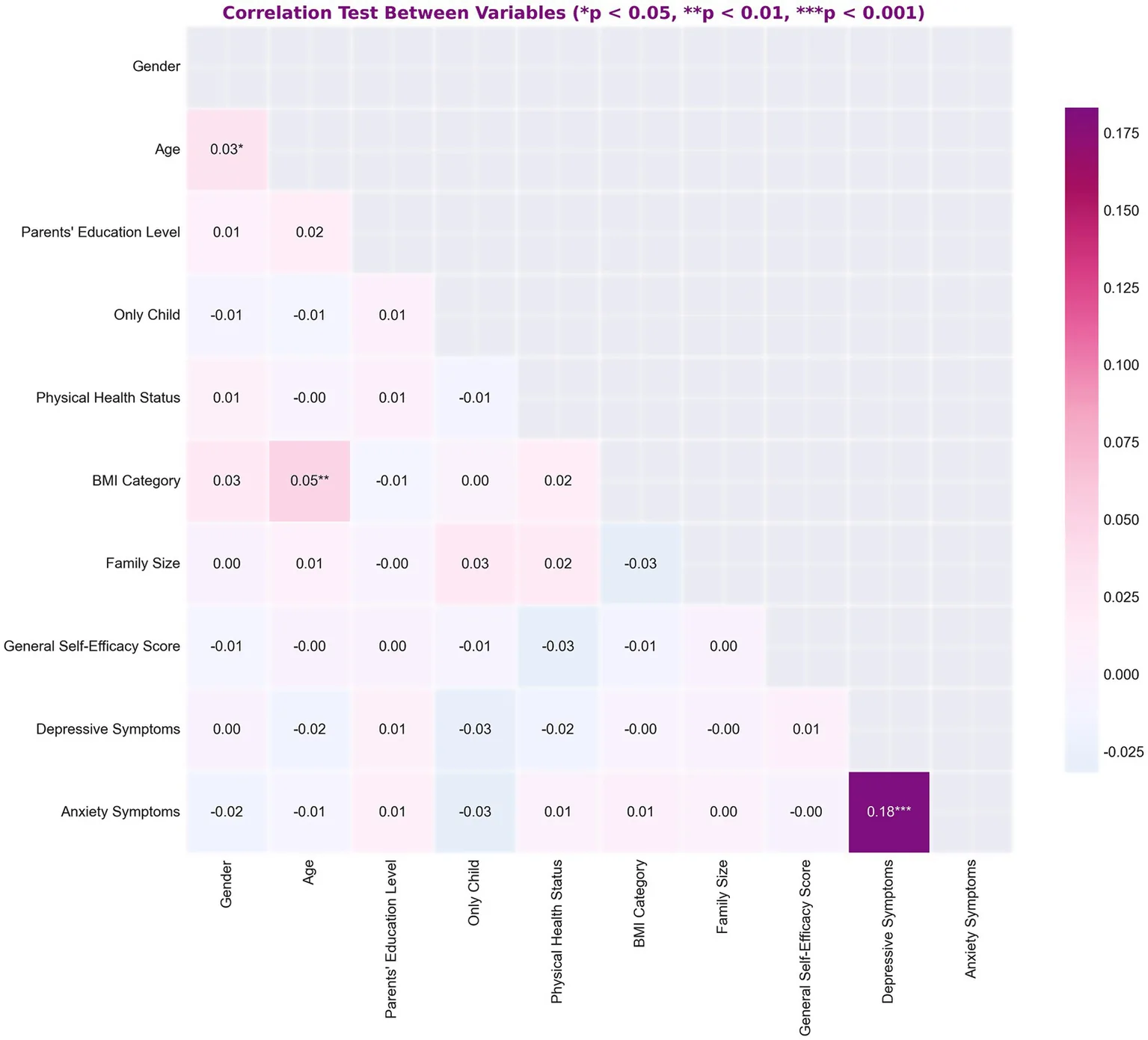
Correlation test between variables.

Supplementary Table 1 shows all variance inflation factor (VIF) values were below 10 (range: 1.001–3.192), confirming no multicollinearity concerns among predictors. Harman’s single-factor test yielded seven components with eigenvalues greater than 1, and the first component accounted for 22.56% of the total variance, well below the 40% threshold, indicating that common method bias was unlikely to threaten the results. Supplementary Table 2 and Figure 2 comprehensively compare the performance of eight machine learning models for predicting smartphone addiction. Linear regression, Ridge regression, and Bayesian Ridge regression demonstrated superior performance with nearly identical R^2^ values (0.478) and the lowest prediction errors (RMSE ≈ 8.66, MAE ≈ 6.96). Figure 2 illustrates that these linear models maintained stable cross-validation performance (CV R^2^ ≈ 0.485, SD = 0.018) and achieved the best model fit according to AIC criteria (4464–4,466). In contrast, ensemble methods showed diminished performance, with Random Forest achieving moderate results (R^2^ = 0.444, RMSE = 8.94) while XGBoost yielded the poorest predictive accuracy (R^2^ = 0.385, RMSE = 9.41) and highest model complexity (BIC = 6,660). The consistency of performance metrics across validation folds suggests that simpler linear models are more suitable for capturing the relationships between psychosocial factors and smartphone addiction in this adolescent population.

**Figure 2:**
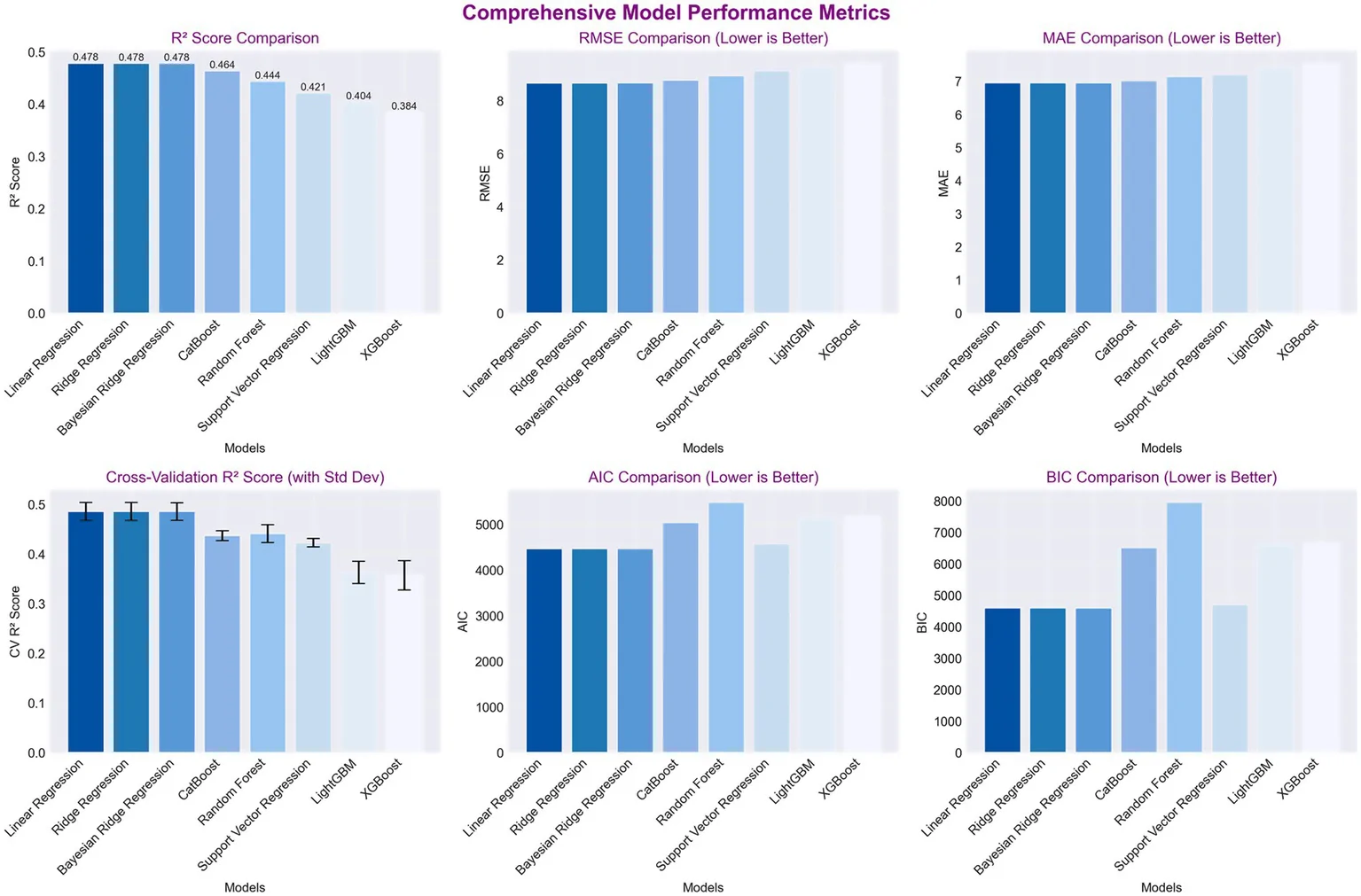
Comprehensive comparison of model performance.

Figure 3 presents a normalized performance heatmap comparing all models across six metrics. Linear regression, Ridge regression, and Bayesian Ridge regression consistently showed superior performance (normalized values approaching 1.0) across all metrics. CatBoost and Random Forest demonstrated intermediate performance (0.6–0.9), while Support Vector Regression, LightGBM, and XGBoost exhibited progressively poorer performance, with XGBoost showing the lowest normalized scores across all evaluation criteria. Figure 4 displays scatter plots of predicted versus actual values for the top four performing models. Linear regression, Ridge regression, and Bayesian Ridge regression showed similar prediction patterns with R^2^ values around 0.478, while CatBoost demonstrated slightly lower predictive accuracy (R^2^ = 0.464).

**Figure 3:**
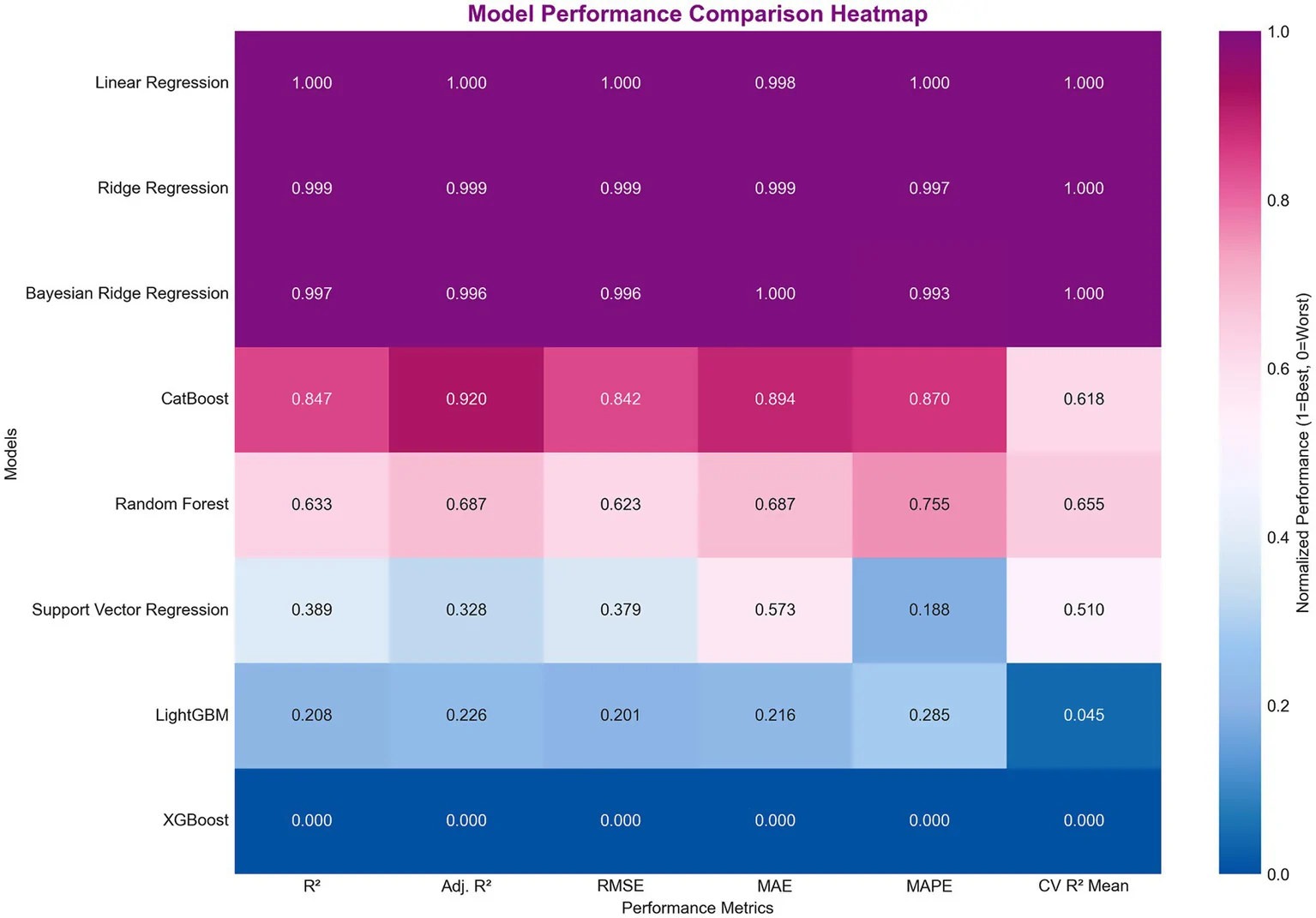
Heatmap of model performance.

**Figure 4:**
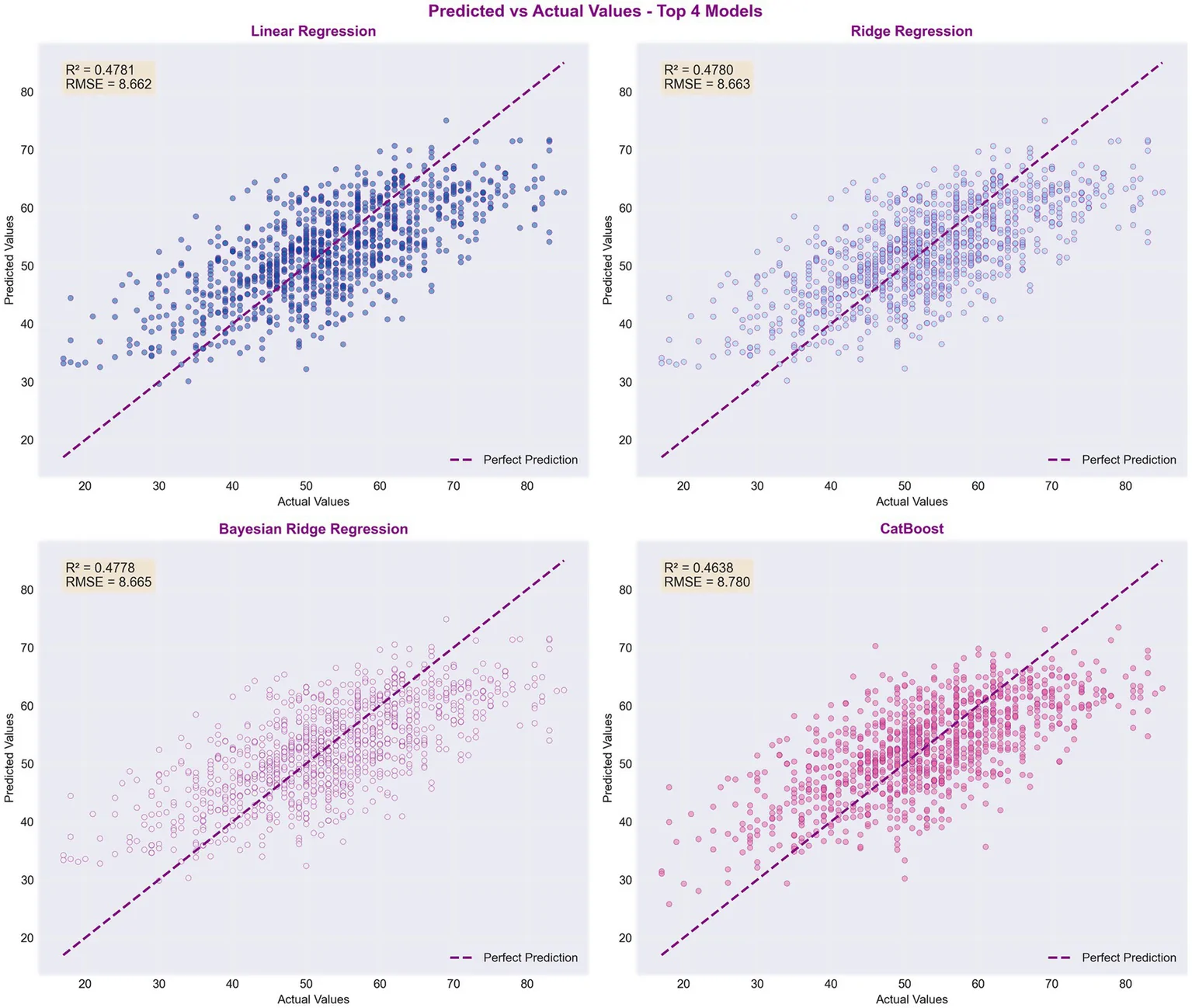
Predicted values vs. actual values.

Supplementary Table 3 and Figure 5 present SHAP-based feature importance analysis for predicting smartphone addiction. Experiential avoidance emerged as the dominant predictor with a SHAP importance value of 5.472, substantially exceeding all other variables. The second tier of important features included depressive symptoms (1.723), BMI category (1.438), age (1.394), and anxiety symptoms (1.337). Perceived social support (0.987) and general self-efficacy (0.909) showed moderate importance, while demographic factors such as family size (0.425) and parents’ education level (0.386) had minimal predictive contribution to the model.

**Figure 5:**
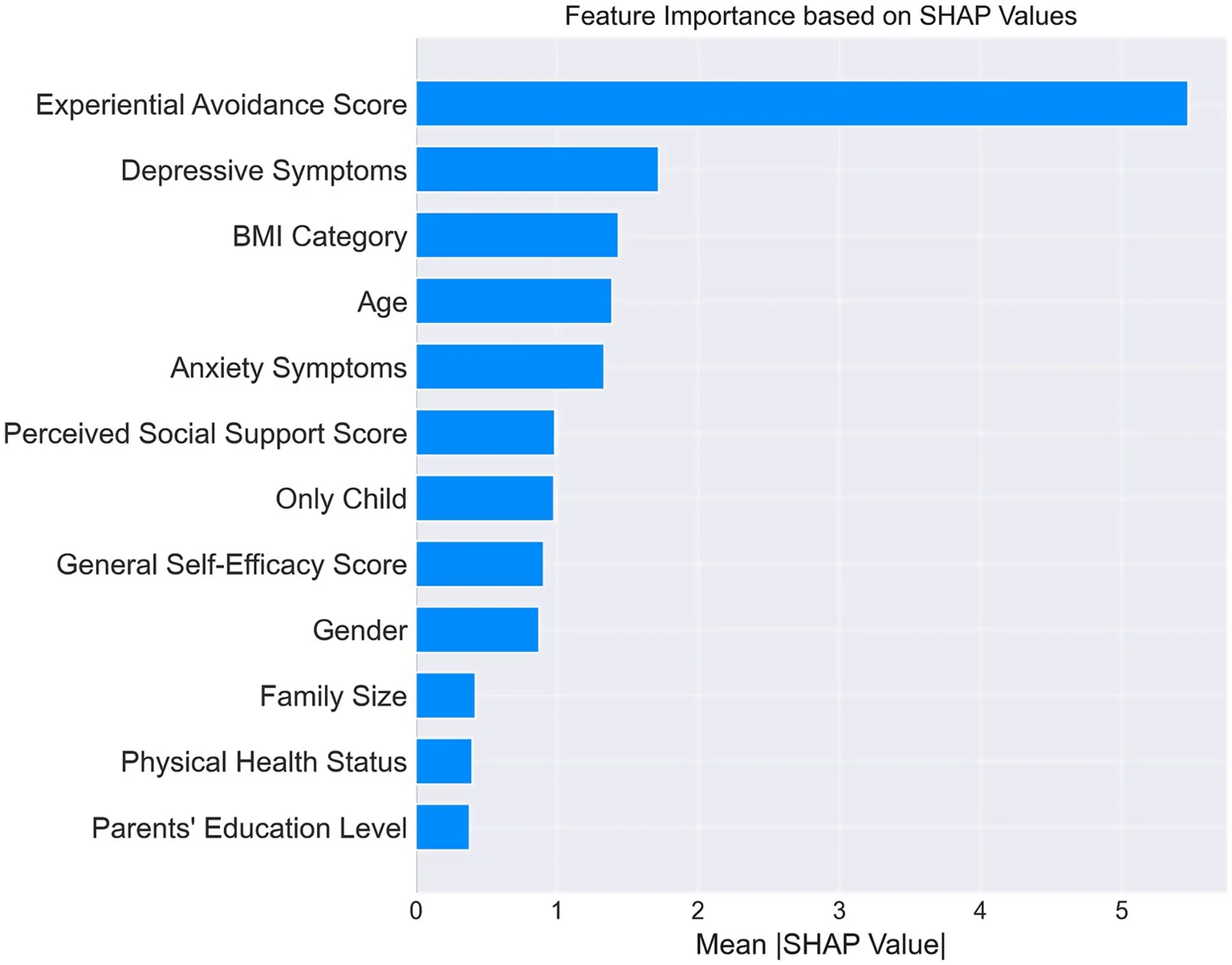
SHAP feature importance bar chart.

Figure 6 illustrates the SHAP value distributions across all features, revealing distinct patterns of association with smartphone addiction. Experiential avoidance showed the widest distribution with a clear positive gradient, where higher scores (red) consistently increased addiction predictions while lower scores (blue) decreased them. Supplementary Figure 2 dependence plots confirm this strong linear relationship for experiential avoidance (SHAP values ranging from −20 to +15). Depressive symptoms, BMI category, and age demonstrated positive but non-linear associations, with threshold effects visible at certain value ranges. In contrast, perceived social support showed a negative relationship, while demographic variables clustered near zero SHAP values, indicating minimal impact on individual predictions despite their inclusion in the model.

**Figure 6:**
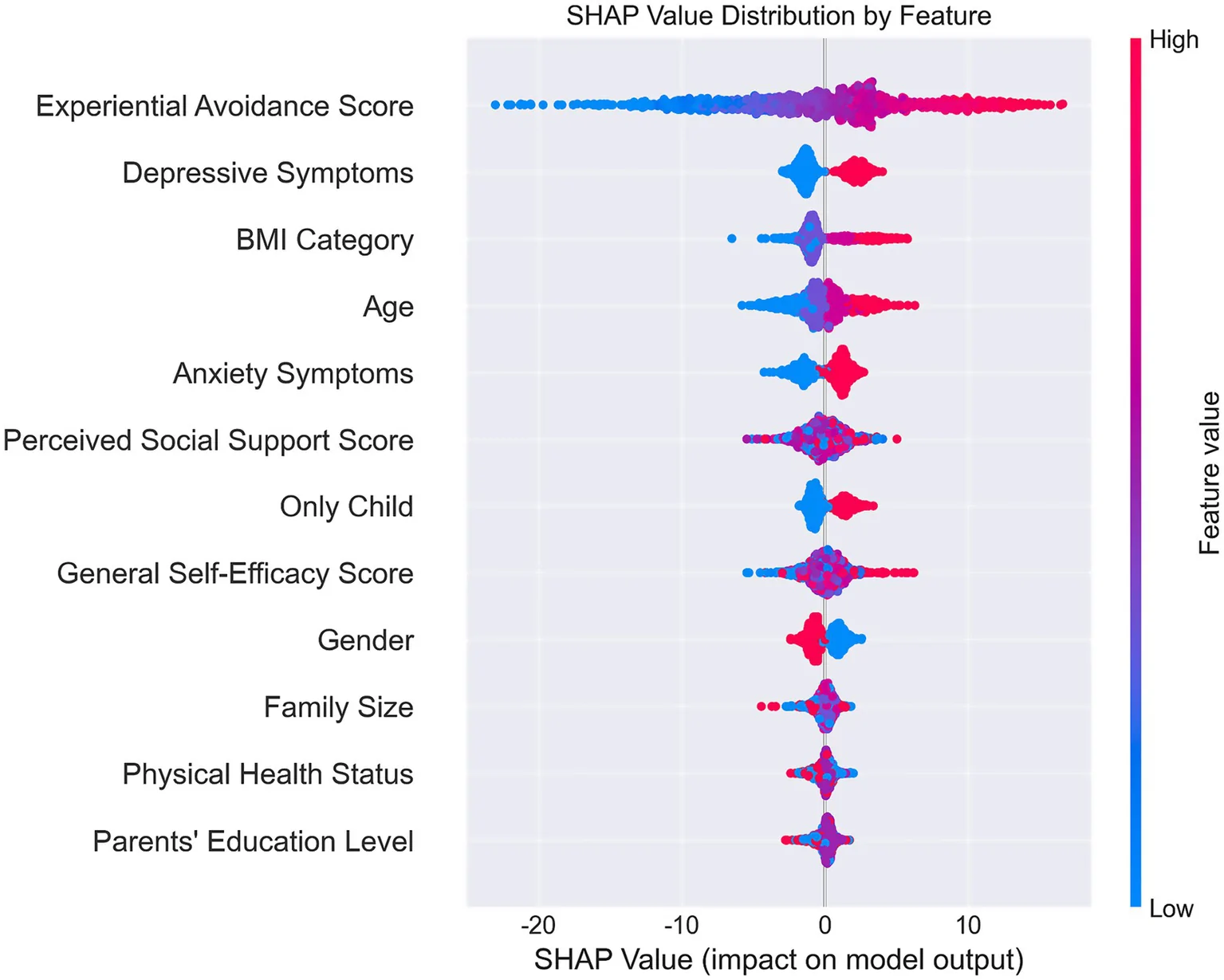
SHAP value distribution plot.

Table 3 and Supplementary Table 4 present the hierarchical regression results examining predictors of smartphone addiction. The baseline model with control variables (age, gender, only-child status, BMI category, parents’ education level, family size and self-rated physical health) explained only 5.6% of variance (R^2^ = 0.056, *F* = 28.784, *p* < 0.001). Adding perceived social support in Model 2 increased explained variance to 10.9% (ΔR^2^ = 0.053, *β* = −0.231, *p* < 0.001). Model 3, incorporating depression and anxiety symptoms, explained 23.6% of variance (ΔR^2^ = 0.127), with both emotional symptoms showing significant positive associations (β depression = 0.255, β anxiety = 0.213, *p* < 0.001). The final model including experiential avoidance dramatically improved prediction, explaining 49.4% of total variance (ΔR^2^ = 0.257, *F* = 303.027, *p* < 0.001), with experiential avoidance emerging as the strongest predictor (*β* = 0.573, *p* < 0.001). Notably, social support’s association with smartphone addiction became non-significant (*β* = 0.020, *p* > 0.05) after controlling for experiential avoidance, suggesting potential mediation effects.

**Table 3:** Hierarchical regression analysis predicting smartphone addiction.

Model	R^2^	Adj. R^2^	ΔR^2^	F	*p*	ΔF	ΔF p
Model 1: Controls	0.056	0.054	0.056	28.784	< 0.001	28.784	< 0.001
Model 2: + Social Support	0.109	0.107	0.053	52.364	< 0.001	205.401	< 0.001
Model 3: + Depression & Anxiety	0.236	0.234	0.127	105.761	< 0.001	284.647	< 0.001
Model 4: + Avoidance	0.494	0.492	0.257	303.027	< 0.001	1738.640	< 0.001

Model 1 entered the control variables (age, gender, only-child status, BMI category, parents’ education level, family size and self-rated physical health). ΔF tests the increment in R^2^ at each step.

Figure 7 and Table 4 present the path analysis examining mediating mechanisms between social support and mobile phone addiction. The total effect of social support on addiction was −0.231 (95% CI [−0.263, −0.200]), decomposed into a non-significant direct effect (0.020, 95% CI [−0.006, 0.047]) and substantial indirect effects (−0.252, 95% CI [−0.275, −0.230]). The primary mediation pathway through experiential avoidance accounted for the largest indirect effect (−0.194, 95% CI [−0.214, −0.175]), representing 83.9% of the total effect. Serial mediation pathways through depression→avoidance (−0.013, 95% CI [−0.018, −0.009]) and anxiety→avoidance (−0.011, 95% CI [−0.015, −0.008]), together with the simple pathways through depression (−0.017, 95% CI [−0.024, −0.012]) and anxiety (−0.016, 95% CI [−0.021, −0.011]), contributed smaller but significant indirect effects. The model explained 49.4% of the variance in smartphone addiction (R^2^ = 0.494, *F*(11, 3,421) = 303.03, *p* < 0.001), indicating that the association between social support and smartphone addiction was almost entirely indirect and carried mainly by experiential avoidance, with emotional symptoms playing secondary mediating roles.

**Figure 7:**
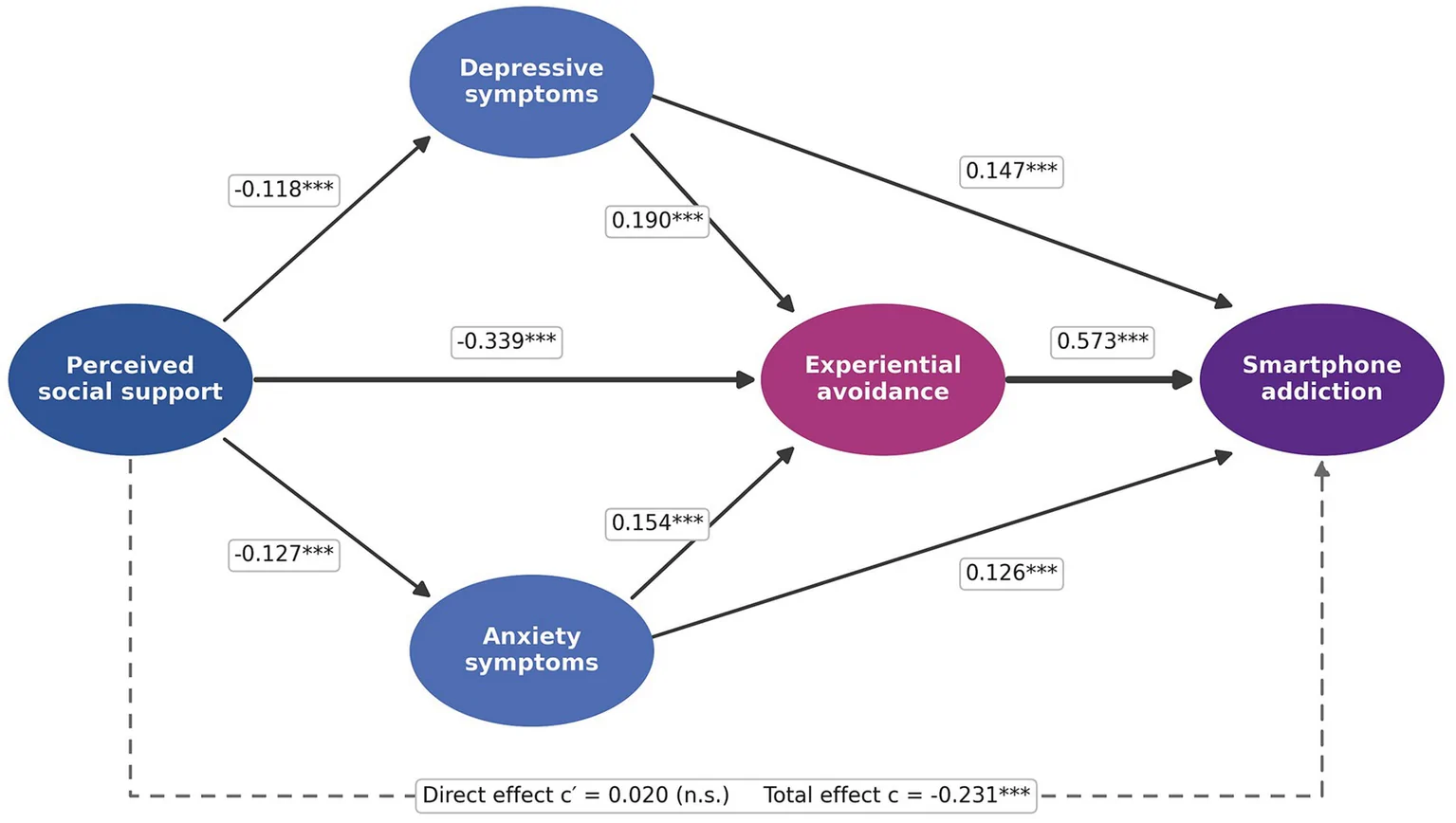
Path model of the associations between perceived social support, depressive and anxiety symptoms, experiential avoidance and smartphone addiction.

**Table 4:** Total, direct and indirect effects of perceived social support on smartphone addiction.

Effect	β	Boot. SE	95% CI
Total effect (c)	−0.231	0.016	[−0.263, −0.200]
Direct effect (c′: SS → SA)	0.020	0.013	[−0.006, 0.047]
SS → Experiential avoidance → SA	−0.194	0.010	[−0.214, −0.175]
SS → Depressive symptoms → SA	−0.017	0.003	[−0.024, −0.012]
SS → Anxiety symptoms → SA	−0.016	0.003	[−0.021, −0.011]
SS → Depressive symptoms → Experiential avoidance → SA	−0.013	0.002	[−0.018, −0.009]
SS → Anxiety symptoms → Experiential avoidance → SA	−0.011	0.002	[−0.015, −0.008]
Total indirect effect	−0.252	0.011	[−0.275, −0.230]

SS, perceived social support; SA, smartphone addiction. Standardized coefficients. Standard errors and 95% percentile confidence intervals are based on 5,000 bootstrap resamples; an interval that excludes zero indicates a significant effect. All equations controlled for age, gender, only-child status, BMI category, parents’ education level, family size and self-rated physical health.

## 4 Discussion

This study employed eight machine learning algorithms to compare prediction performance for smartphone addiction, revealing that linear regression, Ridge regression, and Bayesian Ridge regression achieved optimal performance (R^2^ = 0.478, RMSE = 8.66, MAE = 6.96), outperforming complex ensemble algorithms. Although in other applications ensemble approaches such as Super Learning have shown as good or significantly better predictive performance than linear regression and other methods when used in 13 real datasets, with AUC for models ranging between 0.793 and 0.820 (Acion et al., 2017), the finding that simpler linear models outperformed complex algorithms aligns with recent comparisons showing that standard regression approaches, while not automatically including cross-validation or interactions, can produce comparable or superior predictive ability when the underlying relationships are fundamentally linear (Gowin et al., 2019). In the present data this result most likely reflects the nature of the predictors: the psychosocial variables are summed scale scores that relate to smartphone addiction in a largely additive way, so tree-based ensembles gained little from modeling interactions and non-linearities and paid a price in variance. For these variables, therefore, the added complexity of ensemble methods was not needed to capture the main structure of the data. Experiential avoidance emerged as the primary predictor of smartphone addiction (SHAP value = 5.472), followed by depressive symptoms (1.723), BMI (1.438), age (1.394), and anxiety symptoms (1.337). The association between social support and smartphone addiction was significantly mediated by experiential avoidance, with indirect effects accounting for 83.9% of the total effect. Because the direct association was close to zero and slightly positive, the total indirect effect slightly exceeded the total effect; the percentages reported here should therefore be read as descriptive indicators of the relative size of the pathways rather than as exact proportions.

Our findings align with emerging research linking experiential avoidance to smartphone addiction in adolescents. A recent study found that harsh parenting may be indirectly related to smartphone addiction through both parallel mediation and serial mediation involving depression and experiential avoidance (Lin et al., 2023). Research on 837 junior high school students (ages 12–15) demonstrated that experiential avoidance mediated the relationship between negative school gossip and mobile phone addiction (Xiong et al., 2023). As in Lin et al., the serial pathway through emotional symptoms and experiential avoidance was smaller than the pathway through experiential avoidance alone, suggesting that the two routes are complementary rather than redundant (Lin et al., 2023). Furthermore, experiential avoidance (i.e., attempts to avoid thoughts, feelings, memories and physical sensations) has been associated with excessive smartphone use and social networks, with high scores in maladaptive cognitive-emotion regulation strategies such as self-blame, ruminating and catastrophizing thoughts (Wacks and Weinstein, 2021). In the present study, experiential avoidance emerged as the strongest correlate of smartphone addiction in adolescents, exceeding even depression and anxiety symptoms in relative importance.

Depressive symptoms as the second most important factor corroborates extensive previous research. A meta-analysis revealed moderate but statistically significant correlations between problematic social media use and depression (*r* = 0.273, *p* < 0.001; Shannon et al., 2022). Students with more severe depressive symptoms were independently and significantly associated with higher risk of Internet addiction (OR = 1.07, 95% CI: 1.05–1.09, *p* < 0.001; Xu et al., 2020). The BMI-addiction association found in our study aligns with research showing emotion regulation difficulties, unregulated eating, restrained eating, food addiction, and higher percent body fat were associated with excessive smartphone use among adolescents (Domoff et al., 2020). Age-related findings support longitudinal studies showing that academic stress, social rejection and peer victimization, as well as family dysfunction, are main predictors of smartphone addiction, with effects varying across developmental stages (Crowhurst and Hosseinzadeh, 2024). These findings collectively delineate a multidimensional risk factor profile for adolescent smartphone addiction. After adjustment for the full set of covariates, gender, only-child status and BMI category remained independently associated with smartphone addiction: girls scored higher than boys, in line with the higher levels reported among female adolescents elsewhere (Yıldırım and Çiçek, 2022), only children scored higher than adolescents with siblings, and scores rose across BMI categories. These associations were modest compared with that of experiential avoidance and warrant replication in other samples.

During adolescent development, those adolescents that frequently satisfy their personal motivation by using their smartphones are more likely to become overdependent on these devices, with attachment avoidance confirmed to mediate the association of smartphone addiction with difficulty identifying feelings and externally oriented thinking (Jin et al., 2023). Smartphones serve as uniquely accessible avoidance tools, as both behavioral addictions were associated with impairments in cognitive control related to reward processing (ACC, insula, amygdala) and executive function (DLPFC, frontal, parietal lobes) in adolescents and young adults (León Méndez et al., 2024). Research suggests that ACT can enhance resilience by enhancing psychological flexibility, fostering less rigid and more adaptable responses to challenging situations and emotions (Tayyebi et al., 2024), supporting experiential avoidance as a transdiagnostic factor across different addictive behaviors.

The findings are consistent with the “reality deficit-virtual compensation” framework. The findings resonate with Kardefelt-Winther (2014) compensatory internet use theory, which posits that individuals use digital media to alleviate negative feelings when offline resources are insufficient. Studies show that perceived family support and perceived teacher support significantly positively predict the life satisfaction of college students, while college students generally believe they receive less support than they need, and this difference is associated with more severe depressive symptoms (Rankin et al., 2018). The social compensation hypothesis is supported by research showing people with attachment avoidance tend to have interpersonal functioning of avoidance and apathy; therefore, these people tend to leave their feelings unexplored and not care about the feelings of others (Jauregui and Estevez, 2020). A systematic review found that sources of support varied across life periods, with parental support being most important among children and adolescents (Gariépy et al., 2016). One interpretation of this predominantly indirect association is that social support may need to be internalized, in the form of lower experiential avoidance, before it is reflected in lower levels of smartphone addiction.

Depression and anxiety demonstrated serial mediating effects in the social support-experiential avoidance-smartphone addiction chain. Research has shown that maladaptive cognitive emotion regulation strategies and depression mediate the relationship between shyness and smartphone addiction, with experiential avoidance playing a crucial role (Gao et al., 2025). Network analysis approaches have revealed that higher social support protects people from developing mental disorders through stress-buffering processes, with the protective effect operating through multiple pathways including reduced rumination and improved emotional regulation (Li et al., 2023). Emotional symptoms may heighten experiential avoidance tendencies; relatedly, research has shown that parental smartphone addiction was directly and indirectly related to adolescents’ smartphone addiction through negative parenting attitudes and adolescent aggression (Doo and Kim, 2022). The integration with emotion regulation theory is supported by findings that excessive smartphone use is associated with difficulties in cognitive-emotion regulation, impulsivity, impaired cognitive function, addiction to social networking, shyness and low self-esteem (Wacks and Weinstein, 2021).

This research provides empirical support for the “reality deficit-virtual compensation” framework. ACT programs that mitigate destructive experiential avoidance behaviors, including self-harm behavior and addiction, have shown that after six sessions, participants demonstrated significant improvements in clinical symptoms, frequency of destructive behaviors, and quality of life (Na et al., 2022). Findings indicate consistent effectiveness for ACT and related interventions across various delivery formats, including in-person, group, and internet-based applications, with Intensive ACT groups demonstrating improved depression symptoms and reduced experiential avoidance compared to control groups (Tayyebi et al., 2024). The identification of experiential avoidance as a strong correlate suggests that ACT-based interventions may have significant potential for reducing experiential avoidance and preventing smartphone addiction.

The sample size enhances the reliability of results, providing adequate statistical power for complex modeling. Machine learning approaches in addiction research have shown that models trained on large datasets can achieve robust predictive performance, with studies using samples over 4,000 participants demonstrating stable cross-validation results (Gan et al., 2025). This study tests the complete pathway from social support through experiential avoidance to smartphone addiction in adolescents, addressing a gap in the literature.

## 5 Limitations and future directions

Several limitations should be noted. First, the cross-sectional design precludes causal inference. The ordering of variables in the mediation model was theory-driven, but the data are equally compatible with other orderings: smartphone addiction may itself heighten experiential avoidance and emotional symptoms, and experiential avoidance may precede rather than follow depression and anxiety, as the ACT model would suggest. Unmeasured third variables, such as parenting style or peer relationships, may also account for part of the observed associations. Longitudinal studies have shown that risk factors of smartphone addiction may change over time, with reciprocal relationships between predictors and outcomes (Crowhurst and Hosseinzadeh, 2024), and cross-lagged or intensive longitudinal designs are needed to establish temporal precedence. Second, all measures were self-reported. Harman’s single-factor test suggested that common method variance was not a serious problem, but it cannot rule it out, and self-report data may introduce potential biases, as studies comparing self-reported substance use with biological markers have found discrepancies, suggesting that combining self-report with objective measures improves prediction accuracy (Kim et al., 2025). Objective indicators of smartphone use, such as screen-time logs, would strengthen future work. Third, participants were recruited from schools in a single city, and the clustering of students within schools was not modeled; the findings may therefore not generalize to adolescents in other regions or to those not enrolled in school.

Future research should integrate additional potential variables and physiological indicators for deeper exploration, including neuroimaging markers and real-time behavioral data, and should test whether interventions that reduce experiential avoidance, such as ACT-based programs delivered in schools, are followed by reductions in smartphone addiction.

## 6 Conclusion

This study of adolescents identifies experiential avoidance as a strong correlate of smartphone addiction, with a relative importance substantially exceeding that of depression, anxiety, and demographic factors. The finding that the association between social support and smartphone addiction is almost entirely indirect, and carried mainly by experiential avoidance, is consistent with the “reality deficit-virtual compensation” framework. These results point to experiential avoidance as a promising intervention target, suggesting that ACT-based approaches focusing on reducing avoidance patterns may be more effective than interventions solely addressing social skills or technology behaviors.

## Data Availability

The datasets presented in this article are not readily available because data can be provided upon request. Requests to access the datasets should be directed to food@jlu.edu.cn.
