# Supplementary material for: Experiential avoidance and smartphone addiction in adolescents: a study using multi-model machine learning and mediation analysis
Source: Front Psychol. 2026 Oct 6;17:1860144. doi: 10.3389/fpsyg.2026.1860144 (PMC13680469; doi:10.3389/fpsyg.2026.1860144)
Supplement: Supplementary file 1 [file Supplementary_file_1.docx]

**Supplementary Figure 1: Outlier Detection Box-plots**

**Supplementary Figure 2: SHAP Dependence Plot**

**Supplementary Table 1: VIF Multicollinearity Test**

| **Feature** | **VIF** |
| --- | --- |
| Age | 1.004 |
| General Self-Efficacy Score | 1.001 |
| Perceived Social Support Score | 1.181 |
| Experiential Avoidance Score | 1.275 |
| Gender | 2.031 |
| Parents' Education Level | 2.659 |
| Only Child | 1.504 |
| Physical Health Status | 3.192 |
| BMI Category | 3.010 |
| Family Size | 2.654 |
| Depressive Symptoms | 1.698 |
| Anxiety Symptoms | 2.342 |

**Supplementary Table 2: Comparison of Machine Learning Model Performance**

| **Model** | **R²** | **Adj. R²** | **RMSE** | **MAE** | **MAPE** | **CV R² Mean** | **CV R² Std** | **AIC** | **BIC** |
| --- | --- | --- | --- | --- | --- | --- | --- | --- | --- |
| Linear Regression | 0.4781 | 0.4644 | 8.6622 | 6.9586 | 14.46% | 0.4851 | 0.0180 | 4464.9395 | 4593.1069 |
| Ridge Regression | 0.4780 | 0.4643 | 8.6633 | 6.9580 | 14.46% | 0.4852 | 0.0179 | 4465.1815 | 4593.3489 |
| Bayesian Ridge Regression | 0.4778 | 0.4641 | 8.6649 | 6.9573 | 14.47% | 0.4852 | 0.0178 | 4465.5684 | 4593.7358 |
| CatBoost | 0.4638 | 0.4574 | 8.7800 | 7.0202 | 14.61% | 0.4361 | 0.0098 | 5040.5434 | 6519.3984 |
| Random Forest | 0.4437 | 0.4371 | 8.9428 | 7.1429 | 14.74% | 0.4408 | 0.0180 | 5478.1046 | 7942.8630 |
| Support Vector Regression | 0.4209 | 0.4057 | 9.1247 | 7.2103 | 15.40% | 0.4222 | 0.0085 | 4571.2427 | 4699.4101 |
| LightGBM | 0.4039 | 0.3968 | 9.2572 | 7.4216 | 15.28% | 0.3624 | 0.0221 | 5148.7119 | 6627.5669 |
| XGBoost | 0.3845 | 0.3771 | 9.4072 | 7.5494 | 15.61% | 0.3565 | 0.0297 | 5181.5663 | 6660.4213 |

**Supplementary Table 3: SHAP Feature Importance Analysis**

| **Feature** | **SHAP Importance** |
| --- | --- |
| Experiential Avoidance Score | 5.472 |
| Depressive Symptoms | 1.723 |
| BMI Category | 1.438 |
| Age | 1.394 |
| Anxiety Symptoms | 1.337 |
| Perceived Social Support Score | 0.987 |
| Only Child | 0.983 |
| General Self-Efficacy Score | 0.909 |
| Gender | 0.875 |
| Family Size | 0.425 |
| Physical Health Status | 0.402 |
| Parents' Education Level | 0.386 |

**Supplementary Table 4: Standardized Coefficients from the Hierarchical Regression Models**

| **Model** | **Age** | **Gender** | **Only child** | **BMI category** | **Parents’ education** | **Family size** | **Physical health** | **Social support** | **Depression** | **Anxiety** | **Avoidance** |
| --- | --- | --- | --- | --- | --- | --- | --- | --- | --- | --- | --- |
| Model 1: Controls | 0.154*** | -0.078*** | 0.074*** | 0.139*** | 0.006 | 0.005 | -0.013 | – | – | – | – |
| Model 2: + Social Support | 0.151*** | -0.083*** | 0.076*** | 0.138*** | 0.005 | 0.003 | -0.005 | -0.231*** | – | – | – |
| Model 3: + Depression & Anxiety | 0.157*** | -0.077*** | 0.089*** | 0.136*** | 0.000 | 0.001 | -0.004 | -0.174*** | 0.255*** | 0.213*** | – |
| Model 4: + Avoidance | 0.156*** | -0.079*** | 0.098*** | 0.150*** | 0.013 | -0.004 | -0.002 | 0.020 | 0.147*** | 0.126*** | 0.573*** |

*Note.* Standardized regression coefficients (β). * p < 0.05, ** p < 0.01, *** p < 0.001. Gender: 0 = female, 1 = male; only child: 0 = no, 1 = yes; depression and anxiety: 0 = below cut-off, 1 = at or above cut-off; BMI category, parents’ education, family size and physical health were entered as ordinal scores.
